# Supplementary material for: Digital PBL-CBL teaching method improves students’ performance in learning complex implant cases in atrophic anterior maxilla
Source: PeerJ. 2023 Dec 6;11:e16496. doi: 10.7717/peerj.16496 (PMC10710131; doi:10.7717/peerj.16496)
Supplement: Supplemental Information 3 [file peerj-11-16496-s003.docx]

| **The explanations of the technical terms and jargons in** **“Digital PBL-CBL teaching method improves students’ performance in learning complex implant cases at atrophic anterior maxilla”** | | |
| --- | --- | --- |
| **Item** | **explanation** | **Line number** |
| Prosthetically-oriented placement of dental implants | The concept of " Prosthetically-oriented placement of dental implants " has become the consensus of implantation therapy. The ideal shape and position of the prosthesis are designed according to the facial shape and tooth shape of the patient, and the implant is placed according to the ideal position of the prosthesis to ensure its accurate position and axial orientation. | Line 56 |
| Soft tissue management | Soft management is a step in the dental implant restoration process. In order to achieve better aesthetic results, the soft tissue around the implant should be shaped before the final restoration to form a soft tissue shape similar to the natural gingiva and get a well aesthetic effect. | Line 57 |
| Provisional restoration | Provisional restoration is a type of denture used temporarily before final restoration to improve the contours of the gingival perforation of the implant denture and obtain a more satisfactory esthetic outcome. | Line 57 |
| Final restoration | Final restoration is the final step in the dental implant restoration process.  After the abutment is firmly attached to the implant, a final restoration, such as a  crown, bridge or denture, can be placed on top. Restoration is customized to  fit the patient’s teeth and gums and is designed to look like a natural tooth. | Line 57 |
| Bone augmentation | Bone augmentation, a surgical procedure to increase bone mass, when an implant cannot be done without a certain amount of bone available, it becomes necessary for a periodontal surgeon to stimulate the patient to grow more bone in the area, or to remove bone from one area and place it in the area where the implant will be done. | Line 74 |
| GBR | Guided bone regeneration (GBR) is a bone augmentation method. The theory of GBR technology is to selectively prevent epithelial cells and connective tissue cells from bone defect area through barrier membrane based on different migration rate of various cells, allowing osteoblasts preferentially enter the bone defect area to complete bone induction and regeneration. Meanwhile, bone graft materials are placed in the bone defect area as scaffolds, and guiding osteoblasts and osteocyte to form new bone. | Line 86 |
| Removable teeth-supported prosthetic | Removable teeth-supported prosthetic, which is not fixed permanently in the patient’s oral cavity and can be easily removed by the patient, is a [dental restoration](https://www.sciencedirect.com/topics/medicine-and-dentistry/dental-restoration) helps restore the function and shape of the jaw by replacing one or more missing teeth. | Line 144 |
| Fixed teeth-supported prosthetic | Fixed teeth-supported prosthetic, which can’t be removed or worn by the patient, is a [dental restoration](https://www.sciencedirect.com/topics/medicine-and-dentistry/dental-restoration) used to replace missing teeth and that is permanently attached to adjacent teeth. | Line 144 |
| Soft tissue augmentation | It is a procedure to improve the aesthetic abnormality of the gum and surrounding mucosa and enhance the height and thickness of the soft tissue around the prosthesis or implant. | Line 146 |
| Surgical guide | The surgical guide is a tool to guide the operation based on preoperative 3D simulated diagnostic analysis and surgical planning. | Line 158 |
| 3D titanium mesh | Three-dimensional (3D) printing titanium mesh, which is different from the traditional barrier membrane, can provide good mechanical support and sufficient space for bone regeneration, allowing for the growth and development of new bone. | Line 158 |
| Onlay bone block grafting | Onlay bone block grafting is a bone augmentation method in which the free bone pieces obtained from different parts of the autologous body are fixed under the periosteal of the bone augmentation site, and the mucoperiosteal flap is tightly sutured to promote the healing of the transplanted bone pieces with the original alveolar bone. | Line226 |
