# Supplementary material for: Digital PBL-CBL teaching method improves students’ performance in learning complex implant cases in atrophic anterior maxilla
Source: PeerJ. 2023 Dec 6;11:e16496. doi: 10.7717/peerj.16496 (PMC10710131; doi:10.7717/peerj.16496)
Supplement: Supplemental Information 4 [file peerj-11-16496-s004.docx]

**The Questionnaire Form**

| **Item** | **Opinion of the teaching method** |
| --- | --- |
| 1.I like this approach | Yes □ No □ |
| 2.This approach is efficient | Yes □ No □ |
| 3.This approach decreases extracurricular work | Yes □ No □ |
| 4.This approach makes learning more targeted and more interesting | Yes □ No □ |
| 5.This approach enhances my ability to analyze and solve problems | Yes □ No □ |
| 6.This approach helps me master theoretical knowledge | Yes □ No □ |
| 7.This approach helps me improve clinical skills | Yes □ No □ |
| 8.This approach facilitates clinician-patient communication | Yes □ No □ |
