# Supplementary material for: Digital PBL-CBL teaching method improves students’ performance in learning complex implant cases in atrophic anterior maxilla
Source: PeerJ. 2023 Dec 6;11:e16496. doi: 10.7717/peerj.16496 (PMC10710131; doi:10.7717/peerj.16496)
Supplement: Supplemental Information 5 [file peerj-11-16496-s005.docx]

**调查问卷**

| **项目** | **对教学方法的评价** |
| --- | --- |
| 1.我喜欢该教学方法 | 是 □ 否 □ |
| 2.这种教学方法是有效的 | 是 □ 否 □ |
| 3.这种教学方法减少了课外工作量 | 是 □ 否 □ |
| 4.这种教学方法让学习更具有目标性与趣味性 | 是 □ 否 □ |
| 5.这种教学方法提升了我解决问题分析问题的能力 | 是 □ 否 □ |
| 6.这种教学方法帮助我掌握理论知识 | 是 □ 否 □ |
| 7.这种教学方法帮助我提升了临床技能 | 是 □ 否 □ |
| 8.这种教学方法有助于临床医患交流 | 是 □ 否 □ |
